# Estimating the future global dose demand for measles–rubella microarray patches

**DOI:** 10.3389/fpubh.2022.1037157

**Published:** 2023-01-16

**Authors:** Melissa Ko, Stefano Malvolti, Thomas Cherian, Carsten Mantel, Robin Biellik, Courtney Jarrahian, Marion Menozzi-Arnaud, Jean-Pierre Amorij, Hans Christiansen, Mark J. Papania, Martin I. Meltzer, Balcha Girma Masresha, Desiree Pastor, David N. Durrheim, Birgitte Giersing, Mateusz Hasso-Agopsowicz

**Affiliations:** ^1^MMGH Consulting GmbH, Geneva, Switzerland; ^2^MMGH Consulting GmbH, Zurich, Switzerland; ^3^Independent Consultant, La Rippe, Switzerland; ^4^PATH, Seattle, WA, United States; ^5^Gavi, The Vaccine Alliance, Geneva, Switzerland; ^6^Supply Division, Vaccine Centre, UNICEF, Copenhagen, Denmark; ^7^Global Immunization Division, Centers for Disease Control and Prevention, Atlanta, GA, United States; ^8^Division of Preparedness and Emerging Infections, Centers for Disease Control and Prevention, Atlanta, GA, United States; ^9^Vaccine Preventable Diseases, World Health Organization Regional Office for Africa (AFRO/WHO), Harare, Zimbabwe; ^10^Immunization Unit, Pan American Health Organization (PAHO), Washington, DC, United States; ^11^Medicine and Public Health, University of Newcastle, Callaghan, NSW, Australia; ^12^Immunization, Vaccines and Biologicals, World Health Organization, Geneva, Switzerland

**Keywords:** rubella, microarray patch, microneedle, demand forecast, demand and supply, measles

## Abstract

**Background:**

Progress toward measles and rubella (MR) elimination has stagnated as countries are unable to reach the required 95% vaccine coverage. Microarray patches (MAPs) are anticipated to offer significant programmatic advantages to needle and syringe (N/S) presentation and increase MR vaccination coverage. A demand forecast analysis of the programmatic doses required (PDR) could accelerate MR-MAP development by informing the size and return of the investment required to manufacture MAPs.

**Methods:**

Unconstrained global MR-MAP demand for 2030–2040 was estimated for three scenarios, for groups of countries with similar characteristics (archetypes), and four types of uses of MR-MAPs (use cases). The base scenario 1 assumed that MR-MAPs would replace a share of MR doses delivered by N/S, and that MAPs can reach a proportion of previously unimmunised populations. Scenario 2 assumed that MR-MAPs would be piloted in selected countries in each region of the World Health Organization (WHO); and scenario 3 explored introduction of MR-MAPs earlier in countries with the lowest measles vaccine coverage and highest MR disease burden. We conducted sensitivity analyses to measure the impact of data uncertainty.

**Results:**

For the base scenario (1), the estimated global PDR for MR-MAPs was forecasted at 30 million doses in 2030 and increased to 220 million doses by 2040. Compared to scenario 1, scenario 2 resulted in an overall decrease in PDR of 18%, and scenario 3 resulted in a 21% increase in PDR between 2030 and 2040. Sensitivity analyses revealed that assumptions around the anticipated reach or coverage of MR-MAPs, particularly in the hard-to-reach and MOV populations, and the market penetration of MR-MAPs significantly impacted the estimated PDR.

**Conclusions:**

Significant demand is expected for MR-MAPs between 2030 and 2040, however, efforts are required to address remaining data quality, uncertainties and gaps that underpin the assumptions in this analysis.

## Key points

- The delivery of measles and rubella vaccines with microarray patches (MR- MAPs) could disrupt the immunization landscape.
- We estimated the demand for MR-MAPs between 2030 and 2040 at 4.05 billion doses.
- This analysis will inform the size of investment required to manufacture MR-MAPs.

## Introduction

Prior to widespread vaccination, major epidemics of measles occurred every 2–3 years and measles caused an estimated 2.6 million deaths globally each year, while four babies for every 1,000 live births worldwide were born with congenital rubella syndrome (CRS) (1, 2). Measles and rubella remain a major cause of worldwide morbidity and mortality with an estimated 7.5 million measles cases and more than 60,700 measles-related deaths in 2020, and around 100,000 infants born with CRS each year (1, 3). To eliminate transmission and prevent outbreaks of measles and rubella at least 90–95% of all children must receive both measles and rubella first (MCV1) and second vaccine doses (MCV2) (4).

While the global coverage of MCV1 increased from 72 to 84% between 2000 and 2020, it has stagnated at around 83–85% for the past 10 years (5). MCV2 coverage has been steadily increasing as countries introduce the second dose into their routine schedules but has now also plateaued at around 70% (5). Furthermore, countries and regions are struggling not only to achieve but sustain their measles elimination status through high and widespread levels of vaccination (6). The impact of the SARS-CoV-2 pandemic and its disruptions to routine immunization services and supplemental immunization activities (SIA) have highlighted the precarious situation with regards to measles control and elimination, with the reduction in global MCV1 coverage likely to “fuel a resurgence of measles” (5, 7, 8).

**Figure 1:**
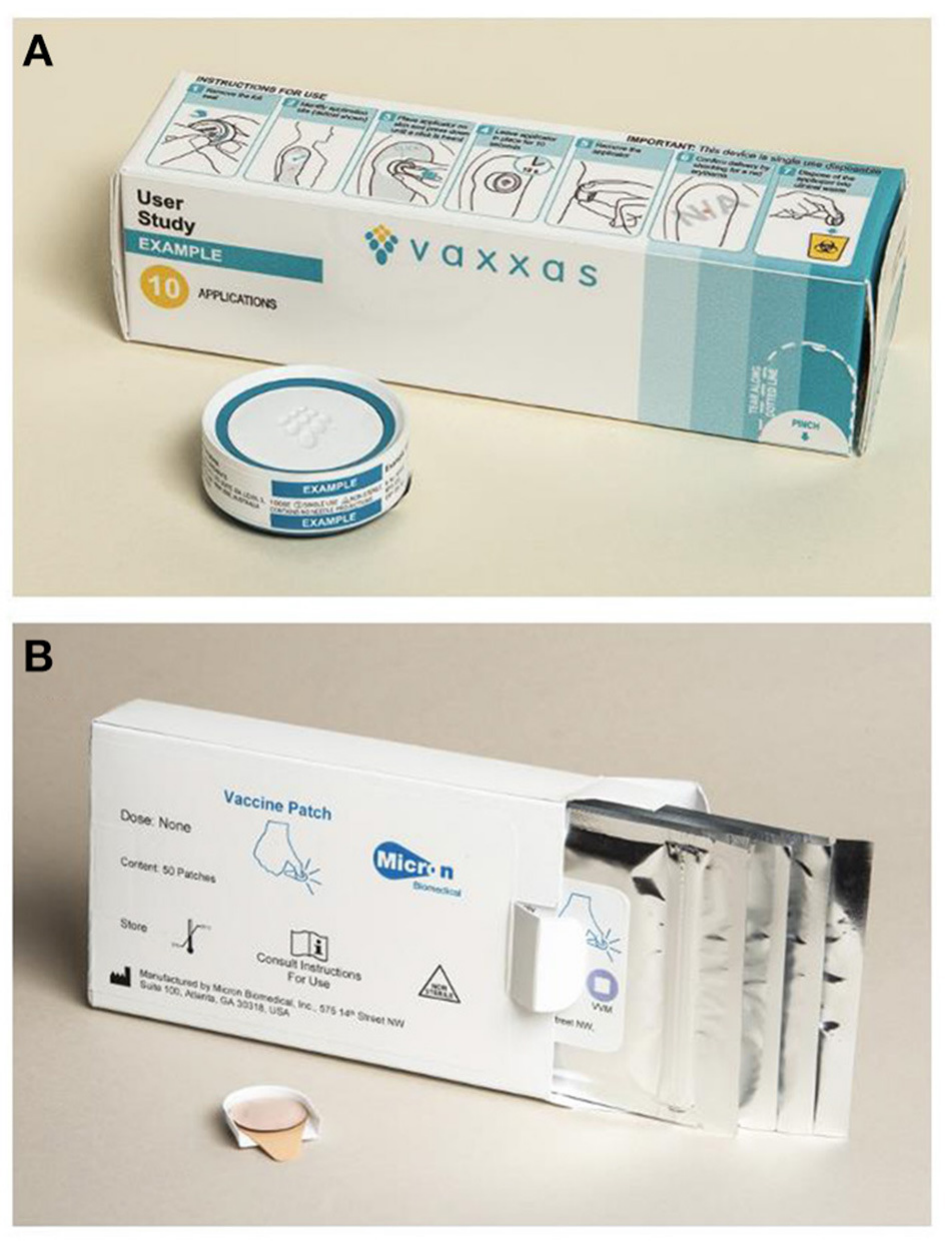
Prototypes of MR-MAPs in development: Vaxxas **(A)** and Micron Biomedical **(B)**. MR-MAP, measles and rubella microarray patches. All images are shared with the companies' permissions.

Efforts to increase coverage and reduce measles and rubella burden could be assisted by the application of innovative technologies such as microarray patches (MAPs) to help reach those communities that are currently under-immunized or completely missed by vaccination (un-immunized). A microarray patch (MAP) consists of hundreds to thousands of tiny projections that deliver vaccine just below the skin surface. Measles-Rubella MAPs (MR-MAPs) are anticipated to be easier to administer than needle-and-syringe (N/S) and be less burdensome on vaccinators and the immunization system, given that they would not require reconstitution, they come as a single dose presentation, have the potential for increased thermostability, and reduced weight (Figure 1). Further, the needleless presentation could address some vaccine hesitancy due to an increasing number of painful injections administered during an immunization session. MAP characteristics required for maximum impact in low- and middle-income countries (LMICs) have been described in the MR-MAP Target Product Profile (9). Given these product characteristics, MR-MAPs are anticipated to be delivered by less trained personal and together with the attributes noted above, facilitate reaching the un- (zero-dose) and under-immunized populations by minimizing delivery challenges. This improved reach is anticipated to ultimately contribute to increasing MCV1 and MCV2 coverage and achieving MR elimination goals. Previous studies have demonstrated MAP acceptability to care givers, its suitability for pediatric use, and preference over N/S administration (10, 11).

While two MR-MAP products have entered Phase I clinical development in 2021 (NCT04394689 and ACTRN12621000820808), they continue to face several hurdles impeding development and slowing licensure timelines. The path to commercialization and uptake is critical since MR-MAP developers or their commercialization partners will need to invest in building and validating MAP manufacturing facilities, and this has not yet been achieved for any vaccine-MAP. Programmatic Doses Required (PDR) are defined as the average estimated number of doses required to meet immunization program needs, whether these are routine or campaign. The PDR includes wastage, which is dependent on the vaccine presentation, and buffer stock. Clarity on the potential demand of PDR and how countries plan to use MR-MAPs could help to inform manufacturing investment decisions. A recent analysis estimates that MR-MAPs could be available to all countries by 2030 (12).

This article summarizes the demand estimates for MR-MAPs between 2030 and 2040. It aims to inform analyses such as cost-effectiveness, willingness to pay, size of manufacturing investment required to manufacture MR-MAPs; as well as other decisions like preparation of appropriate policies, communication or training material to allow for rapid MR-MAP introduction.

## Methodology

To forecast the global MR-MAP demand from 2030 to 2040, a five-step approach was used. This forecast was unconstrained from programmatic, supply, and price perspectives. The methodology described here is a high level, non-technical summary of the approach. A detailed summary of the methodology can be found in Annex A, together with an Excel file “annex.xls” (sheets 1a−8a) that contains data, sources, and all assumptions used in this analysis; and “introdate.xls” that summarizes a model to estimate the year of introduction of MR-MAPs. Additional sensitivity analyses can be found in Annex B.

First, a demand expressed in PDR was estimated for MR vaccines for the years 2030–2040 by leveraging the methodology of the WHO's Market Information for Access to Vaccine's (MI4A) MCV Global Market Study, which contains global MR PDR estimates up to 2030 (13). MI4A is a study that provides a global perspective on vaccine markets, including for measles monovalent, MR, and Measles, Mumps, and Rubella (MMR) or Measles, Mumps, Rubella, and Varicella (MMRV), until 2030 using a population-based methodology, with protocols and assumptions established and reviewed by experts (13). Briefly, the size of the target population, MCV1 and MCV2 coverage, frequency of SIAs, and buffer and stock wastage were used to estimate the MR PDR demand for the years 2030–2040 (Annex A, step 1).

Second, estimates of the number of N/S doses calculated in step 1 that would be replaced by MR-MAPs were made. To achieve this, a set of criteria were identified to predict the year in which a country would adopt MR-MAPs. Then, a market penetration rate of MR-MAPs was applied. Market penetration is defined as the percentage of PDR utilizing MR-MAPs and assumes that MR-MAPs are delivered alongside the N/S presentation. Countries with similar patterns of MR use, or location, were grouped into four archetypes and 16 key countries (Box 1; Annex A, step 2).

Box 1 **Country archetypes.**

**A:** Countries exclusively using MMR or MMRV.

**B:** Countries using MMR or MMRV in routine schedules but utilizing Measles monovalent and MR for SIA activities.

**C:** Countries using MR located in WHO's African and Eastern Mediterranean Region.

**D:** Countries that use M or MR in WHO's Southeast Asian and Western Pacific Region.

**16 Key Countries:** Afghanistan, Bangladesh, Brazil, Chad, Democratic Republic of the Congo, Ethiopia, India, Indonesia, Mozambique, Nigeria, Pakistan, Philippines, South Africa, Tanzania, Uganda, and United States of America.

Third, additional PDR was estimated given the anticipated increased reach of MR-MAPs to zero-dose or under-immunized populations, including remote rural, security compromised, urban slums and missed opportunity for vaccination (MOV) populations. This included defining and developing a set of assumptions concerning definitions, size and vaccine coverage of these additional populations, recommended buffer stocks, and anticipated wastage rates (Annex A, step 3).

Fourth, to understand who would administer MR-MAPs and where, the total MR-MAP PDR from steps 2 and 3 was allocated to the previously defined MR-MAP Use Cases (UCs) using the dimensions of delivery location and service provider (Box 2). The UCs were developed through consultations with National Immunization Programme managers as well as regional and national WHO focal points, and through desk reviews of published and unpublished literature on their potential feasibility and acceptability. These UCs were leveraged to develop an initial estimate of MR-MAP PDR from 2030 to 2040. This exercise estimated the global PDR only for MR-MAPs delivered in a fixed health post or in other settings with limited or no cold chain capacity (UC1, UC2, and UC3/4), while additional research is currently being conducted to estimate PDR for self-administered MR-MAPs (UC5 and UC6), (Annex A, step 4).

Box 2 **Description of the MR-MAP Use Cases.**

**UC1:** Delivery in a fixed health post with full cold chain capabilities by health worker or community health worker.

**UC2:** Delivery in settings with reduced or no cold chain capacity by health worker.

**UC3:** Delivery in settings with reduced cold chain capacity by community health worker.

**UC4:** Delivery in settings with no cold chain capacity by community health worker in their “home” community.

**UC5:** Self-administration with health worker or community health worker supervision.

**UC6:** Self-administration without health worker or community health worker supervision, but may include supervision by another individual (e.g., school teacher, community leader, etc). for monitoring purposes.

Finally, given the level of uncertainty for key assumptions that could significantly impact MR-MAP PDR, three scenarios were explored. The base scenario 1 assumed that MR-MAPs would replace a share of MR doses delivered by N/S, and that MAPs could reach a proportion of previously unimmunised populations. Scenario 2 assumed that MR-MAPs would be first piloted in selected countries in each WHO region prior to wider scale use in countries; and scenario 3 explored earlier introduction of MR-MAPs in countries with the lowest measles vaccine coverage and highest MR disease burden (Annex A, step 5).

Throughout the development of the demand forecast, the Working Group of Experts on MR-MAPs, the MI4A Advisory Group and key country experts were consulted on the methodology and assumptions utilized.

**Figure 2:**
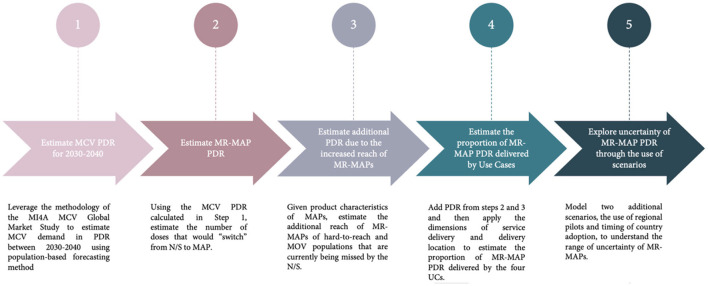
Overview of the five-step approach to develop MR-MAP demand forecast. MR-MAP, measles and rubella microarray patches; MCV, measles containing vaccines; PDR, programmatic doses required; N/S, needle and syringe; MOV, missed opportunities for vaccination; and UCs, use cases.

Figure 2 provides an overview of the five-step process.

## Results

### Scenario 1 (base) results

All numbers presented in this section are approximate. We estimated the PDR for MR between 2030 and 2040 at 4.05 billion doses, with 3.05 billion doses delivered through routine immunization, and ~1 billion doses delivered through SIAs. After applying additional steps including country adoption and market penetration rates (see methods), we estimated the PDR for MR-MAPs between 2030 and 2040 at 1.46 billion doses. We calculated the additional MR-MAP PDR to reach populations not receiving MR vaccines due to MOV or living in hard-to-reach areas (urban slums, remote rural, and security compromised populations) at 0.25 billion doses between 2030 and 2040. This resulted in a total PDR for MR-MAPs between 2030 and 2040 of 1.71 billion doses.

**Figure 3:**
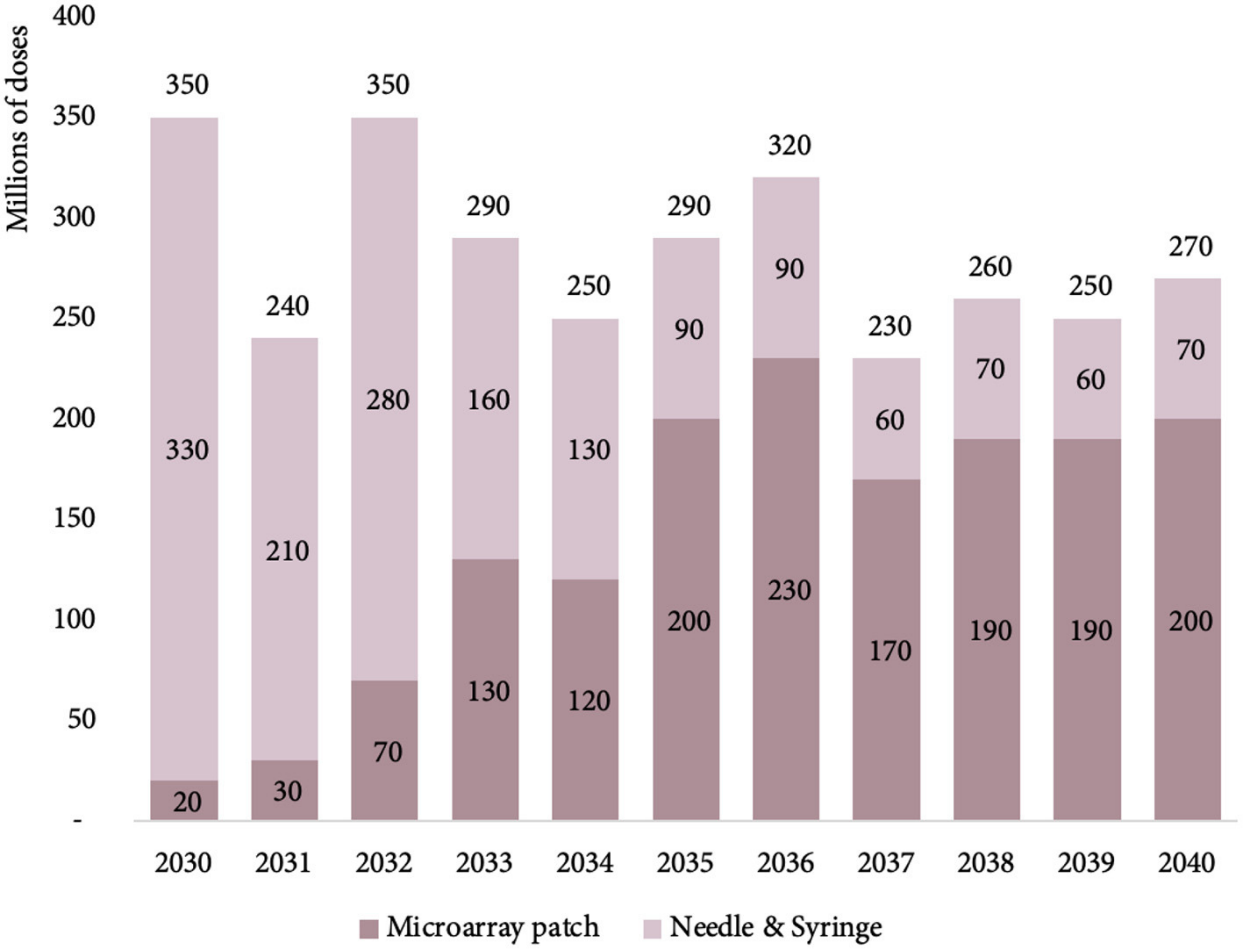
Estimated MR PDR delivered by MAPs and N/S for countries using MR in their national immunization schedules, between 2030 and 2040. MR, measles rubella; PDR, programmatic doses required; MAPs, microarray patches; and N/S, needle and syringe.

The initial MR-MAP PDR in 2030 is estimated at 30 million doses, expected to peak in 2036 at 250 million doses, and level out at 220 million doses in 2040. We estimated that MAPs will deliver a total of 9% (30 million doses) MR PDR in 2030, and steadily increase to ~76% (220 million doses) by 2040 (13). Figure 3 provides an overview of the total number of MR PDR delivered by MAPs and N/S in countries forecasted to use MR in their national immunization schedules.

The majority of the 2030 estimated MR-MAP PDR is from 16 key countries (37%) and Group A countries (50%) with Group B, C, and D accounting for 12% of total PDR. However, by 2040, the percentages are estimated to shift with the 16 key countries accounting for the largest share (61%) of total global PDR, followed by countries in Group C (27%) and countries in Group A, B, and D accounting for 3, 5, and 4%, respectively.

**Figure 4:**
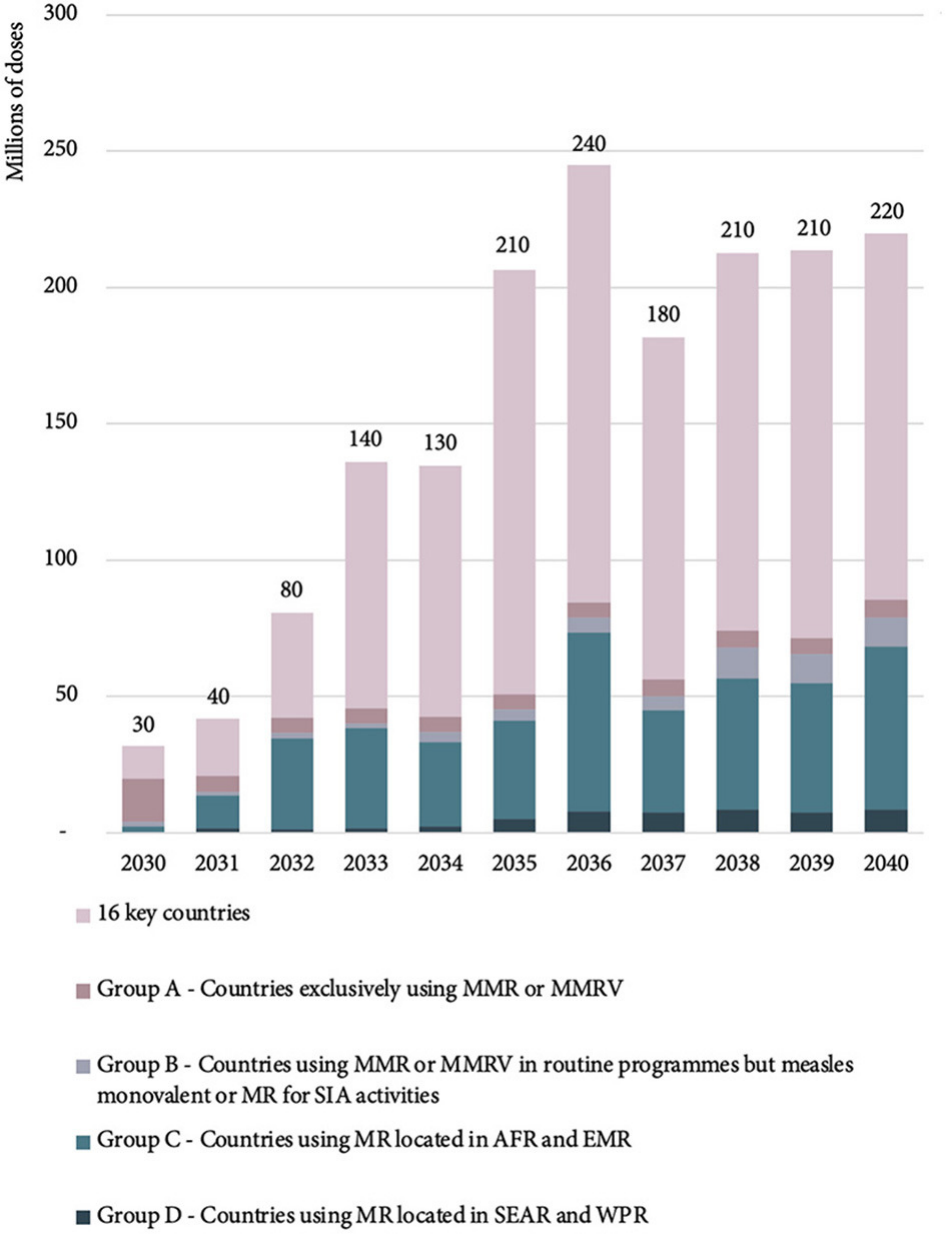
Estimated global MR-MAP PDR—by country group, between 2030 and 2040. MR, measles and rubella; MAP, microarray patches; PDR, programmatic doses required; SEAR, WHO South East Asia Region; WPR, WHO Western Pacific Region; AFR, WHO African Region; EMR, WHO Eastern Mediterranean Region; MMR, measles, mumps, rubella; MMRV, measles, mumps, rubella, varicella; and SIA, supplementary immunization activities.

Figure 4 and Supplementary Table S1 in Annex C provide an overview of the estimated global MR-MAP PDR by country group.

**Figure 5:**
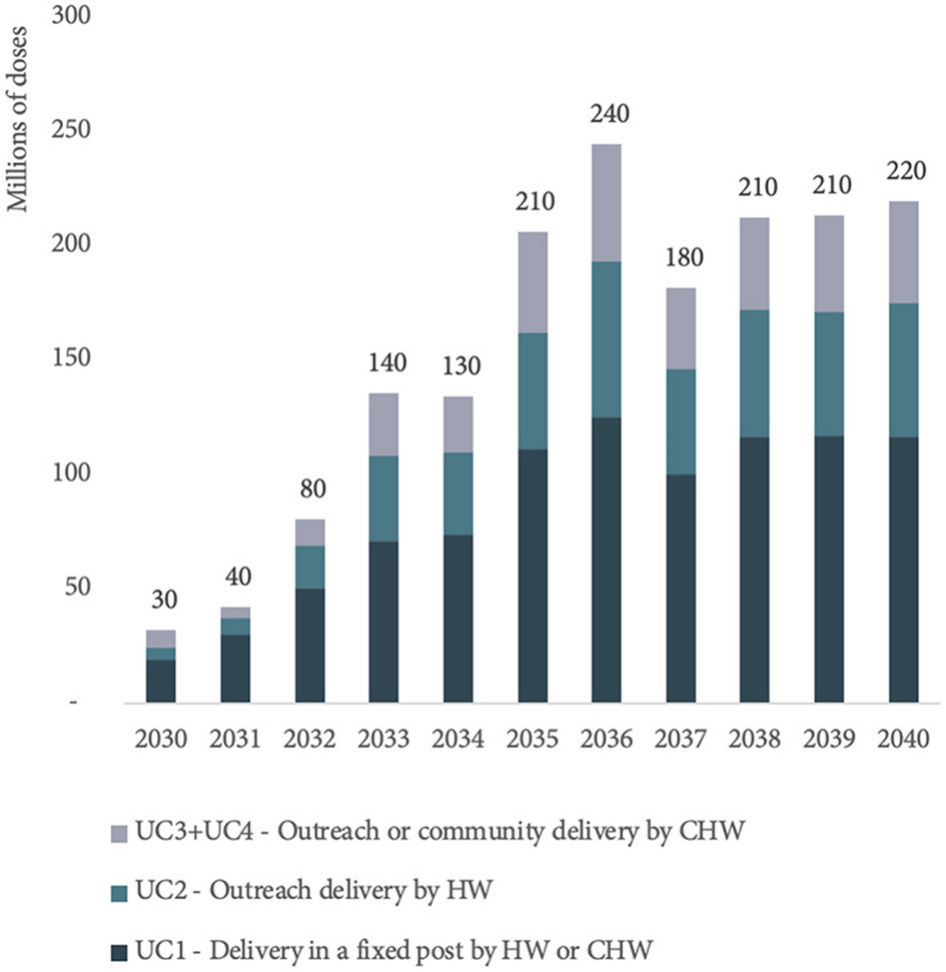
Estimated global PDR for MR-MAPs from 2030 to 2040 by use case. PDR, programmatic doses required; MR, measles and rubella; MAPs, microarray patches; UC, use case; HW, health worker; and CHW, community health worker.

In 2030, 61% of MR-MAP PDR is forecasted to be delivered at fixed health posts with full cold chain capabilities by health workers or community health workers (UC1). The remaining 39% of MR-MAP PDR is anticipated to be used as part of UC2 and UC3/4, i.e., when there is limited or no cold chain capacity and MAPs will be administered by health workers or community health workers. By 2040, 52% of MR-MAP PDR is forecasted to be used in UC1 and 47% in UC2 and UC3/4, showing a slight shift in how doses may be used once most countries have adopted MR-MAPs. Figure 5 and Supplementary Table S2 in Annex C provide an overview of the estimated MR-MAP PDR by UC.

### Exploring uncertainty of global MR-MAP demand with scenarios

In addition to scenario 1 (baseline), two other scenarios were developed to investigate the uncertainty in the trajectory of MR-MAP demand between 2030 and 2040.

Scenario 2 explores the use of regional MR-MAP pilots and results in lower MR-MAP PDR estimates (130 million doses) over the first 5 years compared to the 290 million doses in Scenario 1 (Base) and in a 18% reduction of MR-MAP PDR over the 2030–2040 period.

Scenario 3 explores more accelerated MR-MAP adoption in countries with the greatest needs and estimates an increase in PDR of 186% (830 million doses) in the first 5 years and an overall increase of 21% in PDR over the 2030–2040 period compared to Scenario 1.

**Figure 6:**
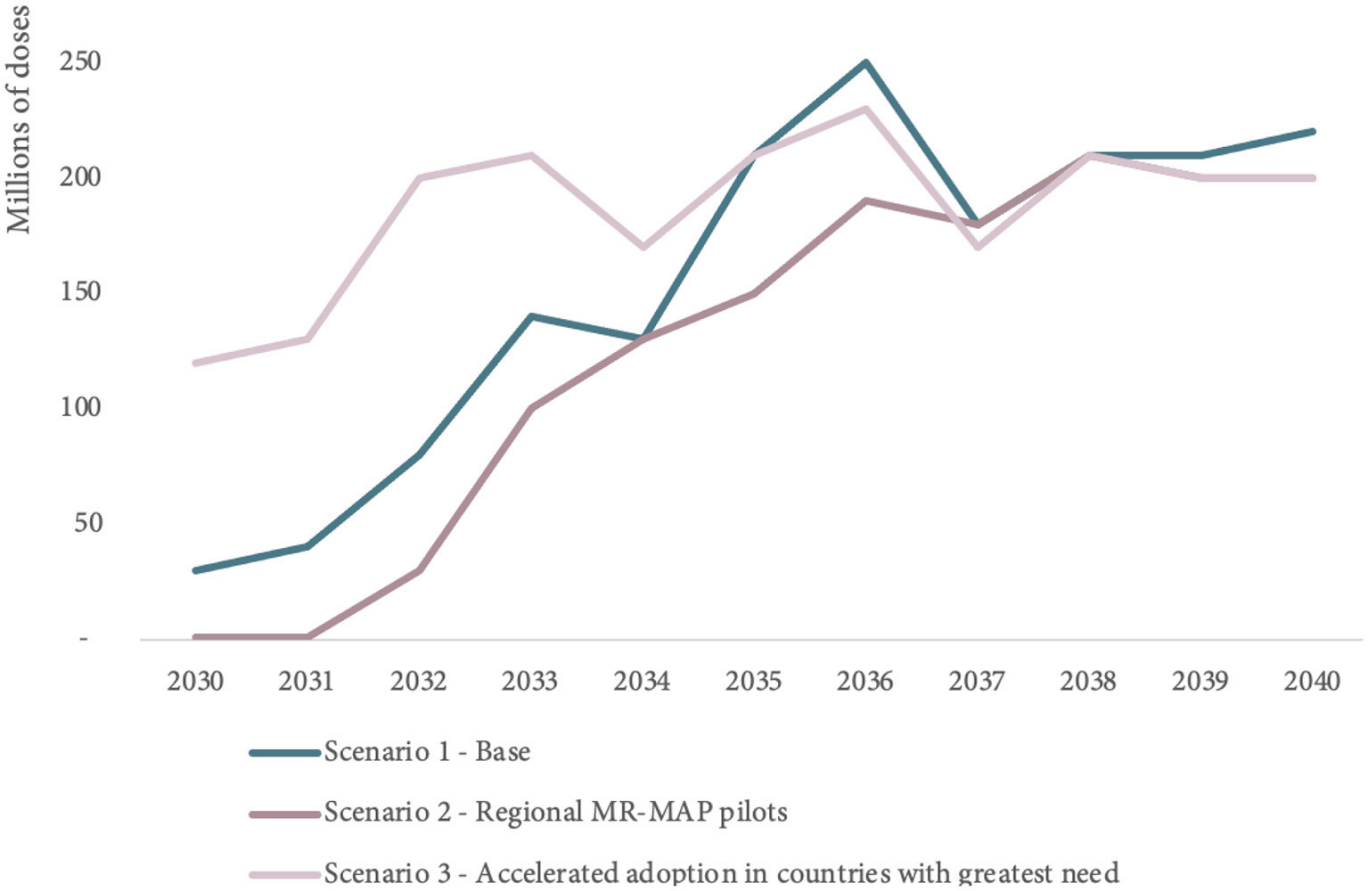
Comparison of global MR-MAP PDR by scenario, between 2030 and 2040. PDR, programmatic doses required; MR, measles and rubella; and MAPs, microarray patches.

While the MR-MAPs PDR estimates for each of the scenarios vary, particularly in the first half of the forecasting period, the PDR estimates ultimately converge toward the latter part of the forecasting period. Figure 6 and Supplementary Table S3 in Annex C provide an overview of the estimated MR-MAP PDR by scenario.

### Limitations

The limitations of this unconstrained demand forecast are largely driven by the limited evidence and data to inform key assumptions, including on how those data may evolve over the next 20 years. We conducted sensitivity analyses to measure the impact of data uncertainty on the estimates of MR-MAP demand between 2030 and 2040 and report the results in Annex B.

In particular the data estimating the size of hard-to-reach populations as well as those prone to MOV that could be reached by using MR-MAPs was scarce with substantial uncertainty in how the situation will evolve over the next 20 years. However, sensitivity analyses revealed that even major changes in the assumptions of estimated population size (e.g., a decrease by 20%) only had a minor impact on the global PDR estimates with a decrease of 1 million doses (see Supplementary Annex B for additional information on the methodology and results of the sensitivity analyses). In contrast, the sensitivity analyses revealed that assumptions around the anticipated reach or coverage of MR-MAPs, particularly in the hard-to-reach and MOV populations, and the market penetration of MR-MAPs significantly impacted the estimated PDR. These assumptions are currently being driven by limited data up to 2020, and gaps were filled by expert opinion and extrapolations made to 2040. Further revisions will require research and consultation to generate a stronger evidence-base.

Other assumptions relating to the delivery of MR-MAPs by less trained individuals (e.g., community health workers) may not be valid due to prevailing and future legal and policy constraints in some countries. These assumptions could also considerably impact global PDR, particularly the split of PDR between the different UCs. Additional evidence is required to understand if and how lesser trained individuals could administer MR-MAPs.

Finally, all assumptions are subject to the perceived benefit, operational feasibility, and cost-effectiveness of switching to MR-MAPs in different country situations. We have assumed that MR-MAPs will have desirable product characteristics based on the Target Product Profile (9). However, uncertainties remain (e.g., thermostability, wear time, vaccine price, ability to reach zero-dose and under-immunized, and impact on cold chain) and these characteristics will likely contribute to country decision making on the adoption and use of MR-MAPs.

As MR-MAPs proceed through the clinical trial phases and the product attributes and operational feasibility become clearer, it will be important to re-visit the applied assumptions and update the demand forecast.

## Discussion

This work represents a first attempt to estimate the unconstrained global PDR of MR-MAPs while considering potential UCs, to inform global discussions and decision making about the investment in, development, introduction, and use of MR-MAPs. As additional information becomes available, the current assumptions should be adjusted to improve the accuracy of the estimates.

We estimate that MR-MAPs PDR will stabilize by 2038 at ~210 million doses per year and will account for ~76% of total MR PDR. In 2040, ~53, 27, and 20% of total MR-MAP PDR will be contributed by UC1, UC2, and UC3/4, respectively (Figure 5). This demand forecast indicates that there will be a substantial and sustainable role for MR-MAPs as part of national immunization programmes. Finally, we estimate that a significant portion of MR-MAP PDR will be used as part of routine immunization (73%) compared to SIAs (27%) by 2040.

Given that MR-MAPs are a new vaccine presentation, a pilot implementation strategy may be the most realistic scenario for their roll-out (Scenario 2). If designed appropriately this strategy could provide valuable evidence on vaccine acceptability and feasibility of uptake and identify best practices to assist countries with their decision making while demonstrating and communicating the benefits of MR-MAPs. Pilot strategies have previously proven useful for countries with the introduction of vaccines using different immunization strategies and varying delivery costs a (e.g., Human papillomavirus (HPV) vaccination demonstration projects and malaria vaccine pilots).

Scenario 3, which forecasts the use of MR-MAPs in countries with the greatest needs, reflects the global public health community's ambition to urgently utilize MR-MAPs as a critical tool to overcome the limitations of current N/S presentations and close coverage gaps. This scenario highlights the potential role that MR-MAPs could play in achieving MR elimination goals.

Additional data and evidence will need to be gathered to refine these estimates, particularly exploring how countries are making decisions on the adoption of MR-MAPs, and how quickly countries could adopt MR-MAPs. We assumed that countries that are using MMR in routine immunization are unlikely to switch to an MR-MAP, despite expected benefits of increasing MR coverage, lacking the mumps antigen. Such assumptions should be evaluated through country consultations. Finally, this demand forecast was considered independent of price assumptions to investigate the full potential of demand, which can ultimately impact Cost of Goods Sold and price. There is a need to further explore how a potential increase in price will be off-set by cost savings in the immunization system and a potentially increased health and economic impact, including the ability of MR-MAPs to reach zero-dose and under-immunized children, which would result in reduced morbidity and mortality. This could play an important role in informing decisions by countries on adopting MR-MAPs.

The demand forecast analysis presented in this article assumes that an MR-MAP will be available in 2030, shortly after the prequalification by WHO in 2029 (12) Several challenges associated with MR-MAP development remain and must be overcome to ensure timely development, these have been summarized by Hasso-Agopsowicz et al. (12) One of the largest barriers to MR-MAP development is the need to invest in a pilot and commercial manufacturing lines that can produce sufficient material for phase 3 trials and beyond. For MR-MAPs to be prequalified in 2029, an investment in the pilot line is required prior to analyzing and reviewing data from phase 2 clinical trials. To date, no funder has committed to take such risk. The MR-MAPs in clinical trials use MR antigen from Serum Institute of India Pvt. Ltd (SIIPL), and there is commitment from SIIPL to provide antigen for the phase 2 clinical study. If a switch of an antigen is required for the phase 3 study, a clinical bridging study should be conducted, further delaying the development timelines (12).

## Conclusions

While MR-MAPs have recently entered Phase I clinical trials, questions on their anticipated use and impact remain (Box 3), which could influence the demand forecast and ultimately affect investment decisions, clinical development strategies and timelines. The use of MAP presentations could potentially change the immunization landscape by addressing some of the current barriers that countries face related to zero-dose and under-immunized children. Thus, it is imperative that these outstanding issues are addressed through open dialogue with countries and manufacturers. As these issues are resolved, the global demand forecasting methodology and assumptions for MR-MAPs will need to be continuously revised to reflect the latest data and information. This can improve the accuracy and reliability of the estimates of MR-MAP demand, which can play an important role in providing confidence to manufacturers of their investments.

Box 3 **Key questions that can impact the demand forecast.**

- What is the anticipated reach or coverage of MR-MAPs in hard-to-reach and MOV populations?
- Can MR-MAPs can be delivered by lesser trained individuals?
- What is the quantification of the perceived benefit, operational feasibility, and cost effectiveness of using MR-MAP in different country situations and contexts?
- Will a potential price increase be offset by other cost savings related to cost savings in logistics, transportation, use of lesser trained individuals, etc?
- Will vaccine purchasers be willing to commit to buy enough MR- MAP doses to provide vaccines manufacturers an acceptable return on investment for developing MAPs or replacing their current N/S product?
- Is it possible to negotiate an advance commitment from vaccine purchasers to encourage rapid commercialization of MR-MAPs?

## Data availability statement

The original contributions presented in the study are included in the article/Supplementary material, further inquiries can be directed to the corresponding authors.

## Author contributions

MK, SM, TC, CM, BG, and MH-A were responsible for the design and implementation of the research. MK and SM analyzed the results. MK and MH-A wrote the article and all authors reviewed the article. MH-A, BG, and MK supervised the work. MH-A and BG secured funding for the research. All authors contributed to the article and approved the submitted version.
