# Supplementary material for: Estimating the future global dose demand for measles–rubella microarray patches
Source: Front Public Health. 2023 Jan 16;10:1037157. doi: 10.3389/fpubh.2022.1037157 (PMC9885039; doi:10.3389/fpubh.2022.1037157)
Supplement: Supplementary file 1 [file Data_Sheet_1.docx]

**Annexes: Estimating the future global dose demand for Measles-Rubella microarray patches**

#

# Supplemental Annex A: Additional information on the methodology and assumptions

## Step 1: Estimate MR PDR for 2030-2040

## Estimate the size of the target population, its characteristics, and immunization strategies

*Data: annex.xls, sheets 2a-2g*

To calculate the size of the future target populations, and the MR PDR for 2030-2040, the medium estimates for the number of surviving infants and nine to 59 month old children were obtained from the United Nations World Population Prospectus (WPP) for years 2030-2040, which provides population estimates for 183 of the 194 UN countries.[13] Two delivery strategies were considered: (i) two MR doses delivered in routine immunization at nine to 12 months and at 15-18 months of age; and (ii) one MR dose delivered as part of supplemental immunization activities (SIAs) to children aged nine to 59 months of age every two to five years until optimal immunity in the target population can be achieved.[4, 14, 15] We assume that all countries in the analysis will have introduced MCV2 by 2030 (e.g., N/S introduction date).[13]

## Forecast MCV1 and MCV2 coverage estimates

*Data: annex.xls, sheets 4a-4b*

The changes in MCV1 and MCV2 coverage between 2030 and 2040 were estimated using the 2019 WHO/UNICEF Estimates of National Immunization Coverage (WUENIC) MCV coverage estimates, leveraging the MI4A methodology, and based on the following assumptions: (i) if coverage is less than 70%, then an annual growth of 3% would be applied; (ii) if coverage was between 70-85% then an annual growth of 1% would be applied; and (iii) finally if coverage was greater than 85%, then an annual growth of 0.5% would be applied.[13] Coverage was capped at 95% or higher if a country has ever achieved greater than 95% in MCV1 or MCV2 coverage. Uptake is a variable that estimates how quickly a country is able to introduce a routine vaccine into its national immunization system. Uptake of MCV1 and MCV2 vaccine was not considered a relevant variable as all countries were forecasted to introduce prior to 2030. [5] The coverage rate and growth were calculated on a yearly basis. A 100% coverage for SIAs was applied to estimate the PDR for SIA; such an assumption was also based on MI4A methodology, which considers the standard practice to procure SIA doses for the entire population.[13] SIAs were assumed to be implemented in one single year (e.g., uptake at 100%) with the exception of countries that have historically implemented SIAs over 2-3 years, including Democratic Republic of Congo, Egypt, Ethiopia, Indonesia, Nigeria, Pakistan, and Philippines. This is in line with the MI4A methodology.[13]

## Forecast the frequency of SIAs

*Data: annex.xls, sheets 8a-8b*

The frequency of the SIAs targeting 9–59-month-old children were forecasted until 2040 based on the forecasted MCV2 coverage using MI4A methodology: (i) if countries had a MCV2 coverage of <60%, they were projected to conduct an SIA every 2 years; (ii) if countries had an MCV2 coverage between 60-80%, they were assumed conducting an SIA every 3 years; and (iii) if countries had a MCV2 coverage of > 80%, SIA were foreseen every 4-5 years.[13] Countries were forecasted to stop conducting SIAs when their MCV2 coverage reached 90% for 3 consecutive years.[4]

## Calculate buffer stock and wastage

*Data: annex.xls, sheet 5*

WHO guidance was followed to estimate buffer stock and wastage rates for the N/S MR vaccine.[16, 17] Annual buffer stock was calculated as 25% of the difference in demand from the current and the prior year of vaccine routine use, negative values were transformed to zero. The following wastage rates were applied for N/S vaccines to be delivered routinely in infants: 1-dose vial 5%, 5-dose vial 15%, and 10-dose vial 40%. The following wastage rates were applied for N/S vaccines to be delivered in SIAs: 1-dose vial 1%, 5-dose vial 10%, 10-dose vial 10%. [16, 17] The wastage rate for MR-MAPs was set at 1% due to their assumed increased thermostability, single dose presentation, and shelf life.[16, 17]

Figure S1 provides an overview of the methodology and assumptions to extrapolate MR routine immunization doses to 2040.

**Figure S1: Methodology and assumptions to estimate MR routine immunization and SIA PDR for 2030-2040 (Step 1).** MR: measles and rubella; SIA: supplementary immunization activities; PDR: programmatic doses required; MI4A: Market Information for Access to Vaccines; UN: United Nations; WPP: World Population Prospectus; WUENIC: WHO/UNICEF Estimates of National Immunization Coverage; MCV1: 1^st^ dose of a measles containing vaccine; MCV2: 2^nd^ dose of a measles containing vaccine.


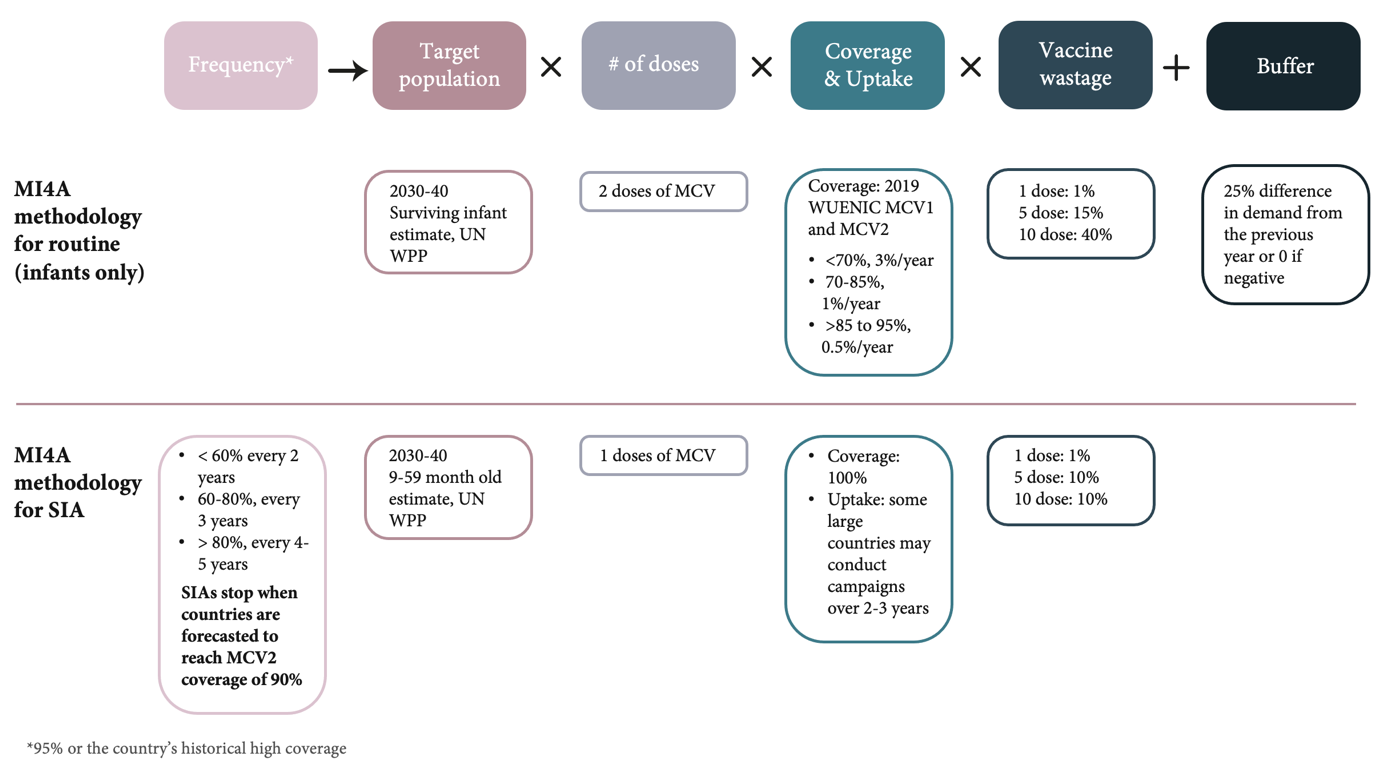


## Step 2: Estimate MR-MAP PDR

## Create country archetypes

*Data: annex.xls, sheet 1a*

The MR-MAP demand forecast utilized a hybrid method where assumptions were developed for four country archetypes, and individually, for 16 key countries. These countries included the ten countries with the largest populations, the ten countries with the largest number of unimmunized children based on MCV1 coverage, and six countries that are judged high priority by the Measles and Rubella Initiative (M&RI) and/or Gavi, the Vaccine Alliance (Gavi). Using these criteria, 16 key countries were identified, and these countries account for approximately 50% of the under 5 year old population.[18] (Figure S2).

**Figure S2 Identification of 16 key countries.** M&RI: Measles and Rubella Initiative; MR: measles and rubella; DRC: Democratic Republic of the Congo; USA: United States of America; MCV1: 1^st^ dose of a measles containing vaccine.


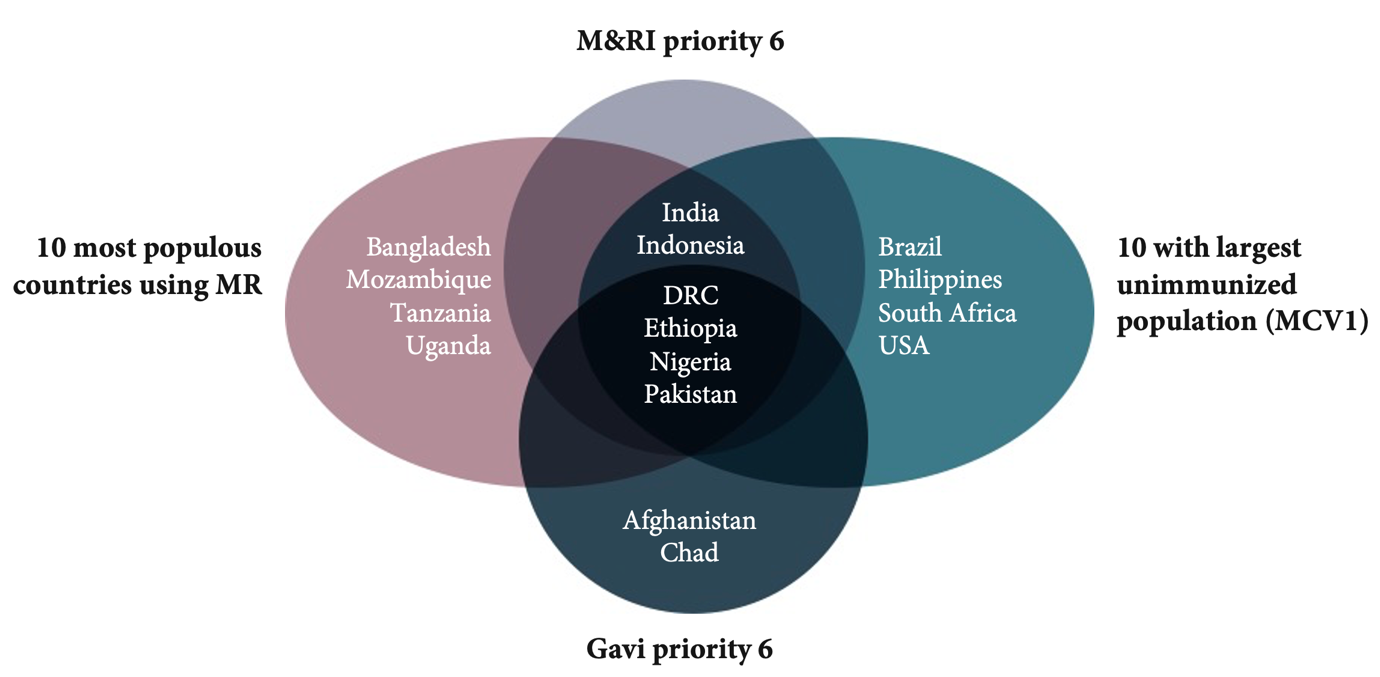


The remaining 167 countries were grouped based on their forecasted MR use and their WHO regions to reflect potential differences in health systems and how vaccines are delivered. This resulted in four country archetypes:

- 1. Countries that exclusively use MMR / MMRV
  2. Countries that use MMR / MMRV in routine immunization but may use MR / M for SIAs
  3. Countries that use M or MR in WHO’s African & Eastern Mediterranean Region
  4. Countries that use M or MR in WHO's Southeast Asian & Western Pacific Region

Where country assumptions were unavailable, the weighted average estimated for the group which included that country was applied.

## Estimate country adoption year of MR-MAPs

*Data: introdate.xls, sheet “intro dates”,*

To estimate the proportion of global MR demand delivered by MAPs, a framework to predict the year of country adoption of MR-MAPs was developed, based on the following three parameters: (i) the year of introduction of MCV2, rubella, pneumococcal conjugate, rotavirus, and human papillomavirus vaccines; (ii) the total disease burden of measles and rubella (2019 reported annual cases and deaths of measles, 2019 measles incidence rate, and 2010 number of estimated rubella cases), and (iii) the percentage of total expenditure on vaccines funded by the government (%) for the last available year or the forecasted Gavi eligibility status in 2030. [19-23]

The predictive framework considers the variables and assigns points distributed by quartiles using the data for each country. For example, the introduction of rotavirus vaccines occurred between 2006 to the present day; thus, those countries that introduced the vaccine within the first quartile or 25^th^ percentile of all years 2006-2022, i.e. years 2006 to 2011, were assigned 4 points, second quartile (i.e., years 2011 to 2014) were assigned 3 points, third quartile (i.e., years 2014-2017) were assigned 2 points, and fourth quartile (i.e., 2017-present day) were assigned 1 point. The quartiles were calculated for each variable with the points assigned in a similar fashion. Then, the scores for each variable were totalled by country, and based on this score, the countries were assigned an adoption year which was equally spread across the 11-year time period of 2030-40. This resulted in ~16 countries adopting MR-MAPs each year. This framework predicts that countries would adopt MR-MAPs earlier if they had a history of early introduction of new vaccines, had a high level of measles and rubella burden, and had sufficient financial resources devoted to immunization or were anticipated to receive donor support. The variables were considered equally for the base estimates.

## Market penetration of MR-MAPs

To estimate the proportion of global MR demand delivered by MAPs, an MR-MAP market penetration rate, varied by country groups, was applied to the total MR PDR calculated from Step 1. In absence of specific market research performed on the topic or of suitable proxies, the assumption of market penetration were based on the following logic: (a) a market penetration of 5% was assumed for Group A as these countries would largely continue using MMR and MMRV for the majority of their programmes, but potentially utilize MR-MAPs to vaccinate special and vulnerable populations, such as migrants; b) a market penetration of 30% was used for Group B countries based on the historical use of MR vaccines in MMR/MMRV countries, which estimated that MR N/S accounted for approximately 30% of total MR demand for these countries[24]; c) a market penetration of 80% was applied for Groups C and D based on the assumption that these countries would unlikely switch fully to MR-MAPs.[24] The logic leveraged the input collected from country immunization experts as part of the interviews. The assumptions were discussed and agreed upon by a group experts.

Figure S3 provides an overview of the methodology and assumptions for Step 2.

**Figure S3: Step 3 Split global MR PDR by presentation and vaccine type.** SIA: supplementary immunization activities; MI4A: Market Information for Access to Vaccines; MR: measles rubella; MR-MAP: measles and rubella microarray patches; N/S: needle and syringe.


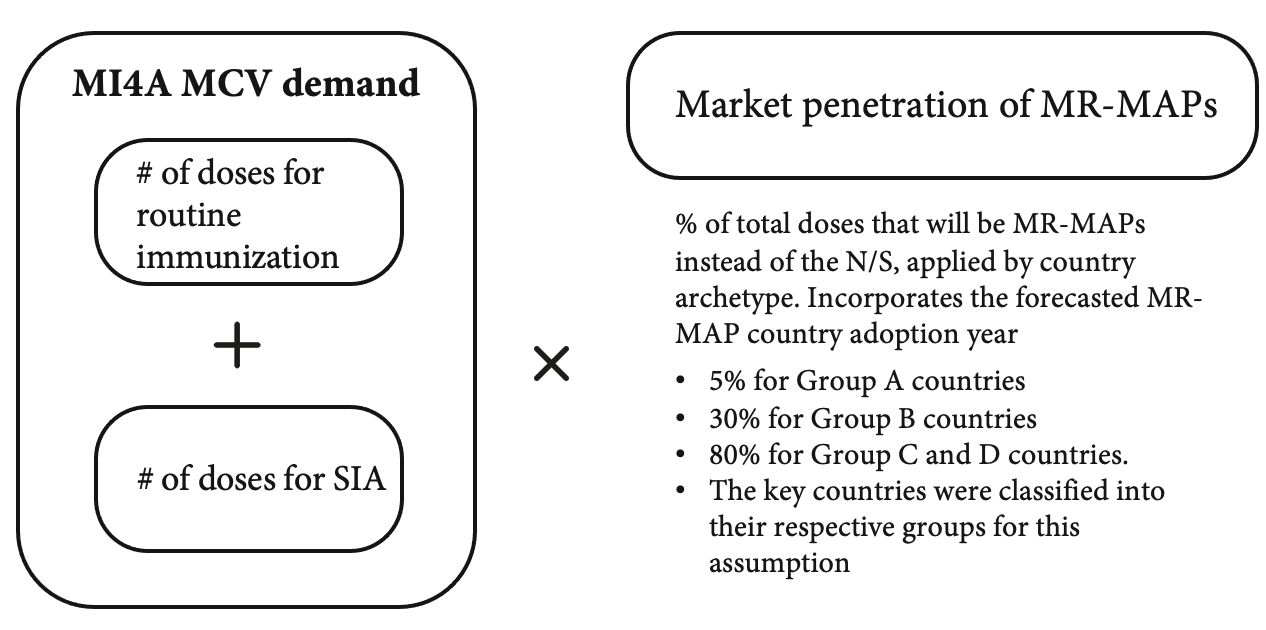


## Step 3: Estimate additional PDR due to increased reach of MR-MAPs

## Characterise the considered target population and immunization strategies

*Data: annex.xls, sheets 3a-3e*

To estimate the full PDR of MR-MAPs, we calculated the size of additional populations that could be reached by MR-MAPs, including wastage and buffer stock. These populations included those who are not receiving MCV vaccines due to missed opportunities for vaccinations (MOV) or living in hard-to-reach areas. We used data from published and unpublished data sources, such as country comprehensive multi-year plans, EPI reviews, Gavi joint appraisal reports, World Bank estimates for population living in urban slums and rural areas, and 2019 United Nations High Commissioner for Refugees estimates for population of concern.[18, 25-30]

To estimate the size of the hard-to-reach populations, the children between 0-2, and 2-15 years old, who lived in urban slums, remote rural, and security compromised settings were included. The relevant data was obtained using the above data sources and then stratified by age based on United Nations World Population Prospectus.[18, 25, 26, 28, 29] The estimates for hard-to-reach populations were held constant for the entire forecasting period given the high level of uncertainty in how these estimates may evolve over the next 20 years.

An MOV refers to any contact with health services by a child or adult who is eligible for vaccination, which does not result in the person receiving one or more of the vaccine doses for which he or she is eligible. The estimated size of the MOV population was calculated as 2% of children under 2 years of age per United Nations World Population Prospectus from 2030-40. This percentage was derived from published MOV literature and validated by expert opinion which considered the timeliness of vaccination and methodology to develop WUENIC estimates to avoid overestimating the MOV population.[18, 28, 29, 31] The MOV percentage was held constant for the forecasting period.

The following immunization strategies were used to reach the MOV and hard-to-reach populations: (i) two doses delivered as part of additional routine immunization for children less than two years of age in MOV and hard-to-reach populations (e.g., security compromised, remote rural, and urban slum populations); and (ii) two doses delivered through SIAs in children between the ages of two to 15 years in hard-to-reach populations.[18, 25-29]

## Forecast coverage of hard-to-reach and MOV populations

*Data: annex.xls, sheets 4c-4d*

For the hard-to-reach and MOV populations, the coverage was estimated based on the expert opinion of members of the Working Group of Experts on MR-MAPs and MI4A Advisory Group. While MR-MAPs were assumed to increase the MR reach in hard-to-reach and MOV populations, their ability to fully address all programmatic barriers was considered unlikely. Hence, for the hard-to-reach and MOV populations, a coverage of 20% was applied in a routine immunization setting for both doses, and 10% was applied for the SIAs. These estimates are conservative given the lack of information on the MAP reach of hard-to-reach and MOV populations. These estimates were also held constant for the forecasting period given the lack of data available to inform this assumption. Uptake of MR-MAPs was assumed to be 100% for both routine and the one-time catch-up campaign of MOV and hard-to-reach populations.

Figure S4 provides an overview of the methodology and assumptions to calculate the additional PDR due to the use of MAPs.

**Figure S4: Methodology and assumptions utilized to calculate additional reach of MR-MAPs in the hard-to-reach and MOV population.** MR-MAPs: measles and rubella microarray patches; MOV: missed opportunities for vaccination; MCV: measles containing vaccines; yr: years.


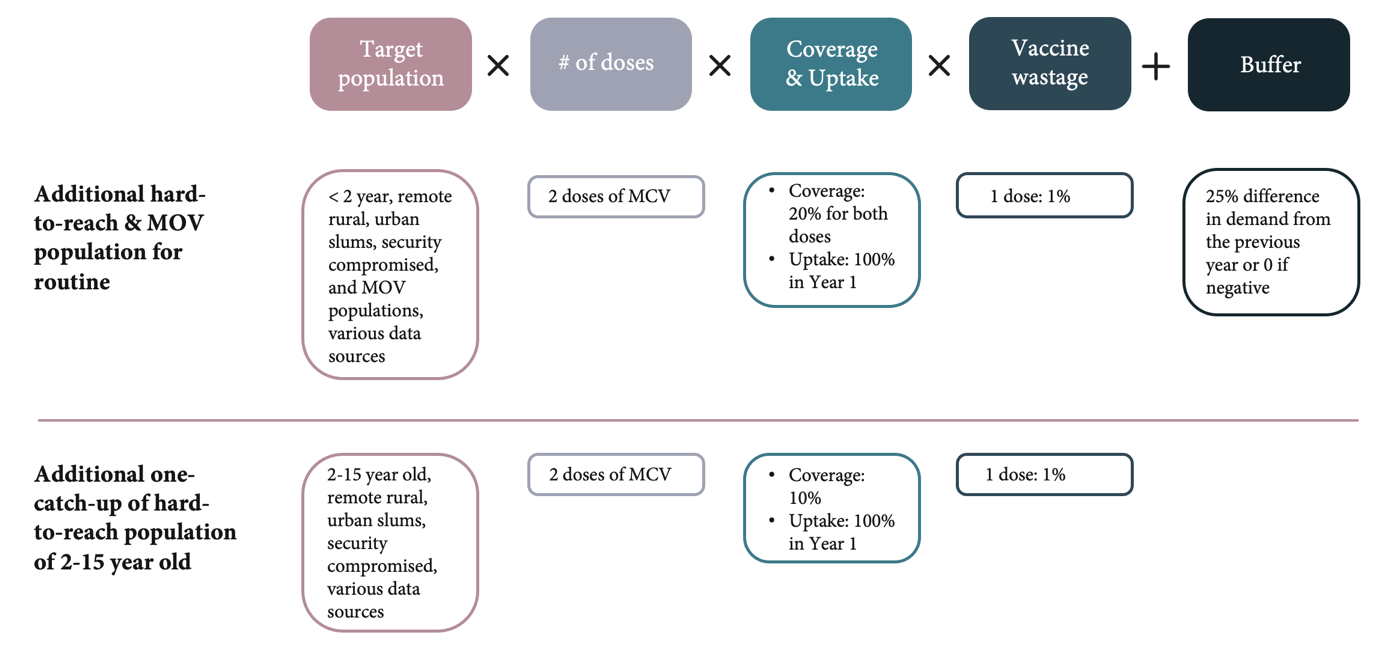


### Step 4: Estimate the proportion of MR-MAP PDR delivered by Use Cases

*Data: annex.xls, sheets 6a-6c*

To estimate where MR-MAPs would be delivered (fixed post with full cold chain capabilities versus limited or no cold chain capabilities) and by whom (health workers, community health workers), we have applied the previously developed Use Cases 1-4 (text box 2) to the MR-MAP PDR developed in step 3. We applied assumptions about the proportion of vaccines delivered in the mentioned locations and by the mentioned personnel. The assumptions relating to the number of nurses or midwives and community health workers were obtained from the Global Health Workforce statistics and supported by unpublished literature, such as country comprehensive multi-year plans. The assumptions related to vaccine delivery in fixed posts or in location with limited or no cold-chain capacity were largely obtained from unpublished literature such as from country comprehensive multi-year plans, Expanded Programme on Immunization (EPI) reviews, Gavi joint appraisal reports, validated during interviews with country EPI managers and immunization focal points. [32] The data was not adjusted for the period 2030-2040, nor it was changed to reflect the introduction of MAPs. Figure S5 provides an overview of the methodology and assumptions.

**Figure S5: Splitting MR-MAP PDR by Use Case.** MR-MAP: measles and rubella microarray patches; PDR: programmatic doses required; HW: health workers; CHW: community health workers; UC: use case.


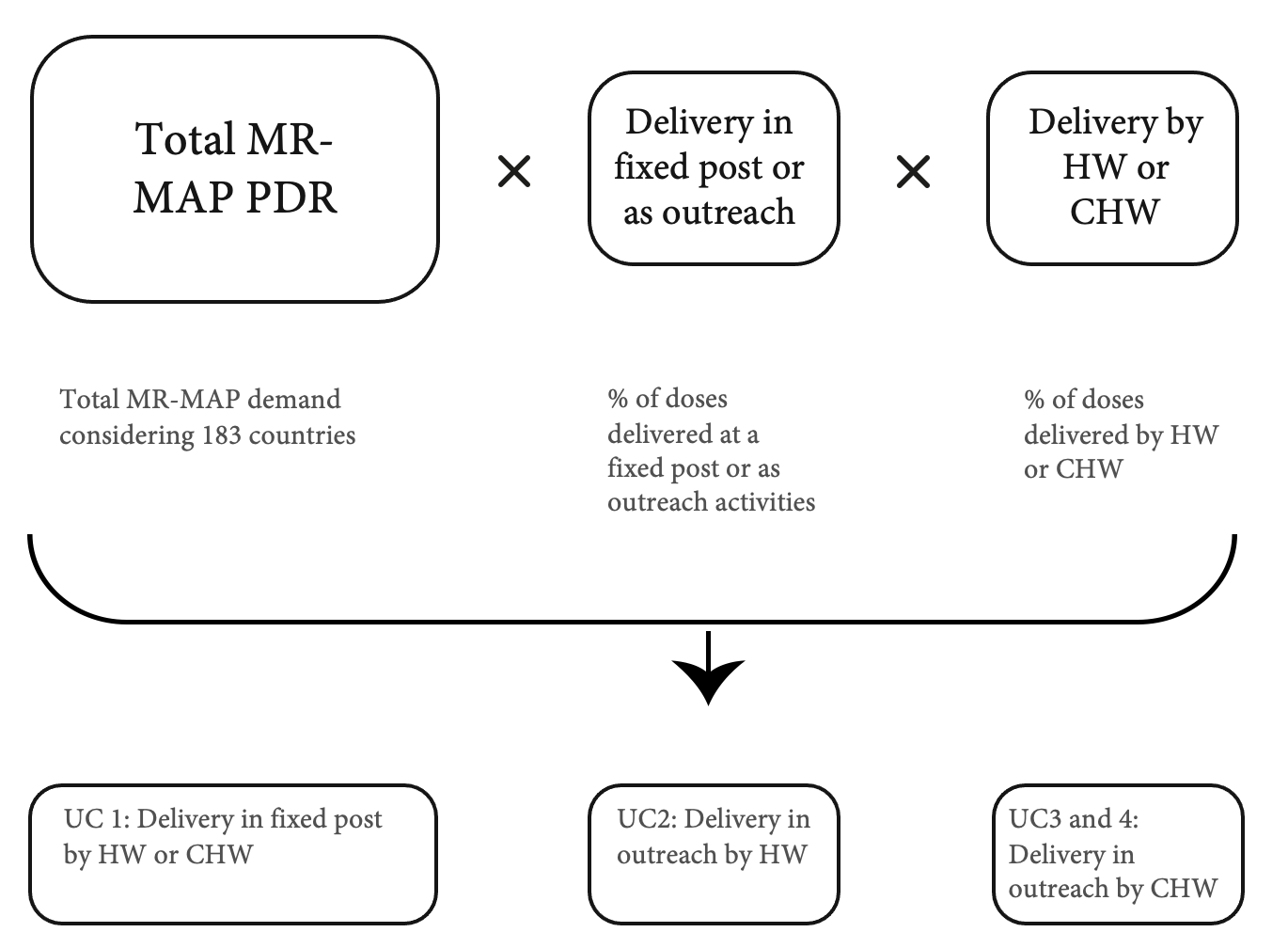


## Step 5: Explore uncertainty of MR-MAP PDR with scenarios

Need data source

Given the high level of uncertainty in how global PDR will evolve for MR-MAPs between 2030-2040, three scenarios were explored (text box 4).

**Text box 4: Description of the scenarios**

- Scenario 1: **Base**: utilizes 5-step demand forecasting methodology described above.
- Scenario 2: **Regional MR-MAP pilots**: Five countries in each WHO region conduct sub-national pilots with MR-MAPs for 2 years prior to national implementation. All other countries do not adopt MR-MAPs until the regional pilots are completed.
- Scenario 3: **Accelerated adoption in countries with the greatest need**: Countries with the lowest MCV1 coverage and highest disease burden for measles and rubella are forecasted to adopt MR-MAPs earlier.

Note that there were no constraints applied to any of the scenarios.

The scenarios were developed based on the input of the Working Group of Experts on MR-MAPs and MI4A Advisory Group. Scenario 1 serves as the **base scenario** and utilizes the forecasting methodology described above to estimate PDR and is described in Steps 1-4. Scenario 2, **regional MR-MAP pilots**, considered if MR-MAPs would be rolled out as ‘sub-national pilots’ in the first five countries in each WHO region targeting only 5% of the surviving infant population for the first two years. These countries would introduce MR-MAPs nationally in year 3 while all other countries would delay the introduction of MR-MAPs until the regional pilots were completed (5 years from the beginning of the pilot). Scenario 3 explored if MR-MAPs were adopted first in countries that had the **greatest need** based on their MCV1 coverage and their disease burden of measles and rubella. This was achieved by utilizing the same predictive framework to forecast adoption year, but instead of the variables being equally weighted, the variables for MCV1 coverage and measles and rubella burden were given twice the weight compared to the other variables. Based on the revised weights, a new adoption year was calculated.

# Supplemental Annex B: Methodology and results of sensitivity analyses

Sensitivity analyses were conducted on specific variables where no or limited data existed or if there were concerns regarding the quality of the data highlighted by the Expert group on MR-MAPs and the MI4A Advisory Group. Four types of sensitivity analyses were conducted on (i) additional hard-to-reach and MOV populations, (ii) proportion of additional hard-to-reach and MOV populations reached by MR-MAPs, (iii) delivery location, and (iv) service provider.

These analyses aimed to identify which variables have the highest impact on the demand forecast so that efforts could be focused on filling the most impactful evidence gaps to improve the accuracy and reliability of the demand forecast.

For simplicity, the variables were considered independently and explored using a plus or minus 20% change. 20% was chosen as the MI4A MCV analysis shows an annual change in total MR routine and SIA PDR of ~22-28%.[13]

The first sensitivity analysis conducted reviewed the estimated hard-to-reach and MOV populations. Based on expert feedback, there were concerns that the definition of these hard-to-reach and MOV populations may either over or underestimate the population and/or there may be overlap between the different populations. Figure S6 provides an overview of the results of the sensitivity analyses for these populations.

**Figure S6: Overview results of sensitivity analyses on hard-to-reach and MOV populations. MOV: missed opportunities for vaccination**

f

The estimated hard-to-reach and MOV populations could have an impact of 1 to 6 million PDR on the overall demand forecast. As this accounts for less than 2% of the total annual PDR once a steady state is reached, it is unlikely that inaccuracies in these population estimates will have significant impact on the overall demand forecast.

Next, we reviewed the proportion of the hard-to-reach and MOV populations that could be reached by a MR-MAP but not a N/S presentation for routine immunization and a one-time catch-up campaign. In contrast, the coverage did have a significant impact on the estimated PDR impacting demand by -84 million doses or by +207 million doses. This highlights the importance in understanding whether MR-MAPs, given their innovative product characteristics, can actually close immunization gaps and reach the zero-dose or under-vaccinated populations. Figure S7 provides the results of this sensitivity analysis.

**Figure S7: Results of the +/-20% sensitivity analysis on the proportion of hard-to-reach and MOV populations that could be reached by a MR-MAP but not a N/S.** MOV: missed opportunities for vaccination; MR-MAP: measles and rubella microarray patches; N/S: needle and syringe; pp: percentage points; m: millions.

Next, we looked at how percentage changes impacted the UC dimensions of delivery location and service provider. While this sensitivity did not change the total estimated PDR for MR-MAPs, it did change how countries would be utilizing the MR-MAPs and the proportion of doses delivered in different UCs.

An increase in the delivery at fixed post by 20% resulted in an increase in UC1 (+41%) with decrease of UC2 and UC3+4 (-38% and -37%, respectively), which focus more on location with limited or no cold chain capacity. In contrast a decrease of 10% delivery in fixed post resulted in a decrease in UC1 (-21%) and increase in UC2 and UC3+4 (+19% for both). The delivery location implies a certain level of stability with the PDR and shows that there is interest for MR-MAPs to be delivered in various locations not only in certain settings.

Finally, considering a +/-20 percent change in whether community health worker could deliver MR-MAPs impacted the PDR between UC2 and UC3+4. If 20% more community health workers are able to deliver MR-MAPs this could create an increase in UC3+4 of ~10% and a decrease in UC2 of -7%. In comparison, if community health workers are unable to deliver MR-MAPs, then this may place more importance on UC2 (+8%) compared to UC3+4 (-12%). Country consultations have indicated that there could be legal and technical hurdles to allowing community health workers to deliver MR-MAPs. These hurdles may reduce a MR-MAP’s value proposition to expand the potential vaccination workforce to reach the zero-dose and under-immunized as it will not allow for differentiation from the N/S presentation.

Given that these sensitivity analyses show ~10% change in the UCs, it highlights the importance of understanding where MR-MAPs will be used and who can deliver MR-MAPs as this could impact the anticipated value of a MR-MAP ultimately affecting investment decisions. Figure S8 provides an overview of the results of the sensitivity analyses on the UC dimensions.

**Figure S8: Results of the +/- 20% sensitivity analysis on UC dimensions of delivery location and service provider.** UC: use case; CHW: community health worker.

# Supplemental Annex C: Summary tables of the demand forecasting results

**Table S1: Estimated programmatic doses required by country archetype and year in millions.** M: measles; MR: measles, rubella; MMR: measles, mumps rubella; MMRV: measles, mumps, rubella, varicella; SIA: supplementary immunization activities; WHO: World Health Organization.

|  | **2030** | **2031** | **2032** | **2033** | **2034** | **2035** | **2036** | **2037** | **2038** | **2039** | **2040** |
| --- | --- | --- | --- | --- | --- | --- | --- | --- | --- | --- | --- |
| 16 key countries | 12 | 21 | 38 | 90 | 92 | 156 | 160 | 126 | 138 | 142 | 134 |
| Group A – Countries exclusively using MMR or MMRV | 16 | 6 | 5 | 5 | 5 | 5 | 6 | 6 | 6 | 6 | 6 |
| Group B – Countries using MMR or MMRV in routine programmes but measles monovalent or MR for SIA activities | 2 | 1 | 2 | 2 | 4 | 4 | 6 | 5 | 12 | 11 | 11 |
| Group C – Countries using M or MR located in WHO’s African & Eastern Mediterranean Region | 2 | 12 | 33 | 37 | 31 | 36 | 66 | 38 | 48 | 47 | 60 |
| Group D – Countries using M or MR located in WHO's Southeast Asian & Western Pacific Region | - | 2 | 1 | 2 | 2 | 5 | 8 | 8 | 8 | 8 | 8 |
| Total | 30 | 40 | 80 | 140 | 130 | 210 | 250 | 180 | 210 | 210 | 220 |

**Table S2: Estimated programmatic doses required by use cases and year in millions. UC: use case.**

|  | **2030** | **2031** | **2032** | **2033** | **2034** | **2035** | **2036** | **2037** | **2038** | **2039** | **2040** |
| --- | --- | --- | --- | --- | --- | --- | --- | --- | --- | --- | --- |
| UC1 – Delivery in a fixed post by health worker or community health worker | 20 | 30 | 50 | 71 | 74 | 111 | 125 | 100 | 117 | 117 | 117 |
| UC2 - Delivery by health worker in locations with limited or no cold chain capacity | 5 | 7 | 18 | 37 | 36 | 51 | 68 | 46 | 55 | 54 | 58 |
| UC3+UC4 - Delivery by community health worker in location with limited or no cold chain capacity | 7 | 5 | 11 | 28 | 25 | 44 | 52 | 36 | 40 | 43 | 45 |
| Total | 30 | 40 | 80 | 140 | 130 | 210 | 250 | 180 | 210 | 210 | 220 |

**Table S3: Estimated programmatic doses required by scenario and year in millions.** MR-MAP: measles and rubella microarray patches.

|  | **2030** | **2031** | **2032** | **2033** | **2034** | **2035** | **2036** | **2037** | **2038** | **2039** | **2040** |
| --- | --- | --- | --- | --- | --- | --- | --- | --- | --- | --- | --- |
| Scenario 1 - Base | 30 | 40 | 80 | 140 | 130 | 210 | 250 | 180 | 210 | 210 | 220 |
| Scenario 2 - Regional MR-MAP pilots | 1 | 1 | 30 | 100 | 130 | 150 | 190 | 180 | 210 | 200 | 200 |
| Scenario 3 - Accelerated adoption in countries with greatest need | 120 | 130 | 200 | 210 | 170 | 210 | 230 | 170 | 210 | 200 | 200 |

Bibliography

4. World Health Organization. Measles vaccines: WHO position paper. Weekly Epidemiological Record 2017; 92(17): 205-27.

5. WHO/UNICEF coverage estimates for 1980-2020. Geneva, 2021.

13. Global Market Study, Measles-containing vaccines. Geneva: World Health Organisation, Market Information for Access to Vaccines (MI4A) Initiative, 2020.

14. Gavi The Vaccine Alliance. Measles and measles-rubella vaccine support. Accessed August 13.

15. World Health Organization. Rubella vaccines: WHO position paper. Weekly Epidemiological Record 2020; 95(27): 306 - 24.

16. How to calculate vaccine volumes and cold chain capacity requirements. Geneva, Switzerland: World Health Organization, 2017.

17. Vaccine Wastage Rates Calculator. Geneva, Switzerland: World Health Organization, 2021.

18. United Nations, Department of Economic and Social Affairs, Division P. World Population

Prospects 2019. Geneva, Switzerland, 2019.

19. Global Burden of Disease Collaborative Network. Global Burden of Disease study 2019 (GBD 2019) Results.

20. World Health Organization. Immunization Data, vaccine introduction. Accessed May.

21. World Health Organization. Immunization data, provisional measles and rubella data. Accessed May.

22. World Health Organization. Immunization expenditure. Accessed May.

23. Vynnycky E, Adams EJ, Cutts FT, et al. Using Seroprevalence and Immunisation Coverage Data to Estimate the Global Burden of Congenital Rubella Syndrome, 1996-2010: A Systematic Review. PLoS One 2016; 11(3): e0149160.

24. Summary Measles-Rubella Supplementary Immunization Activities, 2000-2020. World Health Organization, October 2020.

25. Rural population. In: Prospects UNPDsWU. New York: World Bank, 2018 Revision.

26. Population living in slums (% of urban population). World Bank.

27. Affairs - Population Division of the U. N. Department of Economic and Social. 2018 Revision of World Urbanization Prospects. 2018.

28. Magadzire BP, Joao G, Shendale S, Ogbuanu IU. Reducing missed opportunities for vaccination in selected provinces of Mozambique: A study protocol. Gates Open Res 2017; 1: 5.

29. Sridhar S, Maleq N, Guillermet E, Colombini A, Gessner BD. A systematic literature review of missed opportunities for immunization in low- and middle-income countries. Vaccine 2014; 32(51): 6870-9.

30. End of the year Population of Concern. United Nations High Commissioner for Refugees 2020.

31. The percentage of children aged 12–23 months who have received at least one dose of measles-containing vaccine in a given year. Global Health Observatory. Geneva: WHO, 2019.

32. Global Health Workforce Statistics. Geneva: World Health Organization, 2018 update.
